# PERK in POMC neurons connects celastrol with metabolism

**DOI:** 10.1172/jci.insight.145306

**Published:** 2021-09-22

**Authors:** Zhenyan He, Linh Lieu, Yanbin Dong, Sadia Afrin, Dominic Chau, Anita Kabahizi, Briana Wallace, Jianhong Cao, Eun-Sang Hwang, Ting Yao, Yiru Huang, Jennifer Okolo, Bo Cheng, Yong Gao, Ling Hu, Kevin W. Williams

**Affiliations:** 1 Department of Neurosurgery, Affiliated Tumor Hospital of Zhengzhou University, Zhengzhou, Henan, China.; 2 Center for Hypothalamic Research, Department of Internal Medicine, The University of Texas Southwestern Medical Center at Dallas, Dallas, Texas, USA.; 3 Institute of Gastroenterology and; 4 Science and Technology Innovation Center, Guangzhou University of Chinese Medicine, Guangzhou, Guangdong, China.; 5 Division of Pediatric Endocrinology, Department of Pediatrics, UCLA Children’s Discovery and Innovation Institute, David Geffen School of Medicine at UCLA, Los Angeles, California, USA.; 6 State Key Laboratory of Genetic Engineering, School of Life Sciences, Fudan University, Shanghai, China.; 7 Laboratory Department, Affiliated Hospital of Binzhou Medical College, Shandong, China.

**Keywords:** Endocrinology, Neuroscience, Diabetes, Leptin, Obesity

## Abstract

ER stress and activation of the unfolded protein response in the periphery as well as the central nervous system have been linked to various metabolic abnormalities. Chemically lowering protein kinase R–like ER kinase (PERK) activity within the hypothalamus leads to decreased food intake and body weight. However, the cell populations required in this response remain undefined. In the current study, we investigated the effects of proopiomelanocortin-specific (POMC-specific) PERK deficiency on energy balance and glucose metabolism. Male mice deficient for PERK in POMC neurons exhibited improvements in energy balance on a high-fat diet, showing decreased food intake and body weight, independent of changes in glucose and insulin tolerances. The plant-based inhibitor of PERK, celastrol, increases leptin sensitivity, resulting in decreased food intake and body weight in a murine model of diet-induced obesity (DIO). Our data extend these observations by demonstrating that celastrol-induced improvements in leptin sensitivity and energy balance were attenuated in mice with PERK deficiency in POMC neurons. Altogether, these data suggest that POMC-specific PERK deficiency in male mice confers protection against DIO, possibly providing a new therapeutic target for the treatment of diabetes and metabolic syndrome.

## Graphical abstract

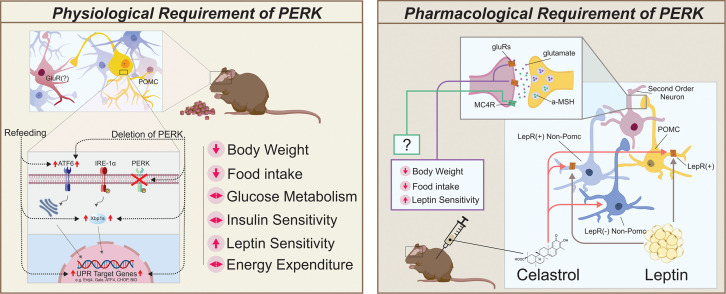

## Introduction

ER stress and the unfolded protein response (UPR) have been linked to several physiological and pathological conditions (1–9). Protein kinase R–like ER kinase (PERK) is part of the ER-anchored receptor signaling network that is involved when the UPR is activated in response to ER stress. When activated, PERK oligomerizes and initiates phosphorylation of eukaryotic initiation factor 2α (Eif2α), thereby downregulating protein translation and preventing accumulation of misfolded proteins in the ER (10).

PERK deficiency in humans is associated with Wolcott-Rallison syndrome (WRS), which results in decreased growth and permanent neonatal or early infancy insulin-dependent diabetes (11). Global mutations of the gene encoding PERK, *Eif2ak3*, in murine models exhibit similar traits to those of WRS within 4 weeks after birth (12–15). In particular, impairment in PERK-dependent phosphorylation of Eif2α in mice leads to decreased body weight, elevated blood glucose levels, and reduced serum insulin (16). Within the central nervous system, the chemical compound celastrol increases leptin sensitivity, resulting in marked improvements in the body weight of diet-induced obesity (DIO) mice and enhancement of leptin-induced hypophagia by reducing ER stress in the hypothalamus via selectively reducing PERK phosphorylation (17). While Liu and colleagues have investigated the effect of celastrol in reducing PERK phosphorylation in the hypothalamus and improving metabolism, the requirement of PERK in mediating the effects of celastrol has yet to be established (17). Moreover, while there is growing evidence that PERK/Eif2α signaling regulates proper body weight maintenance and glucose metabolism in various tissues, including the hypothalamus, the neuronal cell type(s) involved in this response remains undefined.

In the present study, the hypothesis that PERK in proopiomelanocortin (POMC) neurons regulates metabolism was directly tested in mice that were selectively deficient for PERK in POMC neurons. Changes in diet-induced body weight as well as glucose and insulin tolerances were assessed. We also examined the requirement of PERK in POMC neurons in the celastrol-induced improvements of leptin sensitivity and body weight.

## Results

### PERK deficiency in POMC neurons protects against DIO.

In order to test the requirement of PERK in POMC neurons for regulation of energy balance, we generated mice that were deficient for PERK in POMC neurons (POMC-cre:PERK^loxp/loxp^ mice). Notably, there was a marked decrease in PERK mRNA levels within the hypothalamic arcuate nuclei of POMC-cre:PERK^loxp/loxp^ mice when compared with those in littermate controls [PERK, 7 degrees of freedom [t(7)] = 2.490, *P* < 0.05; Supplemental Figure 1; supplemental material available online with this article; https://doi.org/10.1172/jci.insight.145306DS1] This appeared specific to the arcuate nucleus, as there were no differences in PERK mRNA levels in the hindbrains and livers of POMC-cre:PERK^loxp/loxp^ mice (Supplemental Figure 1).

**Figure 1:**
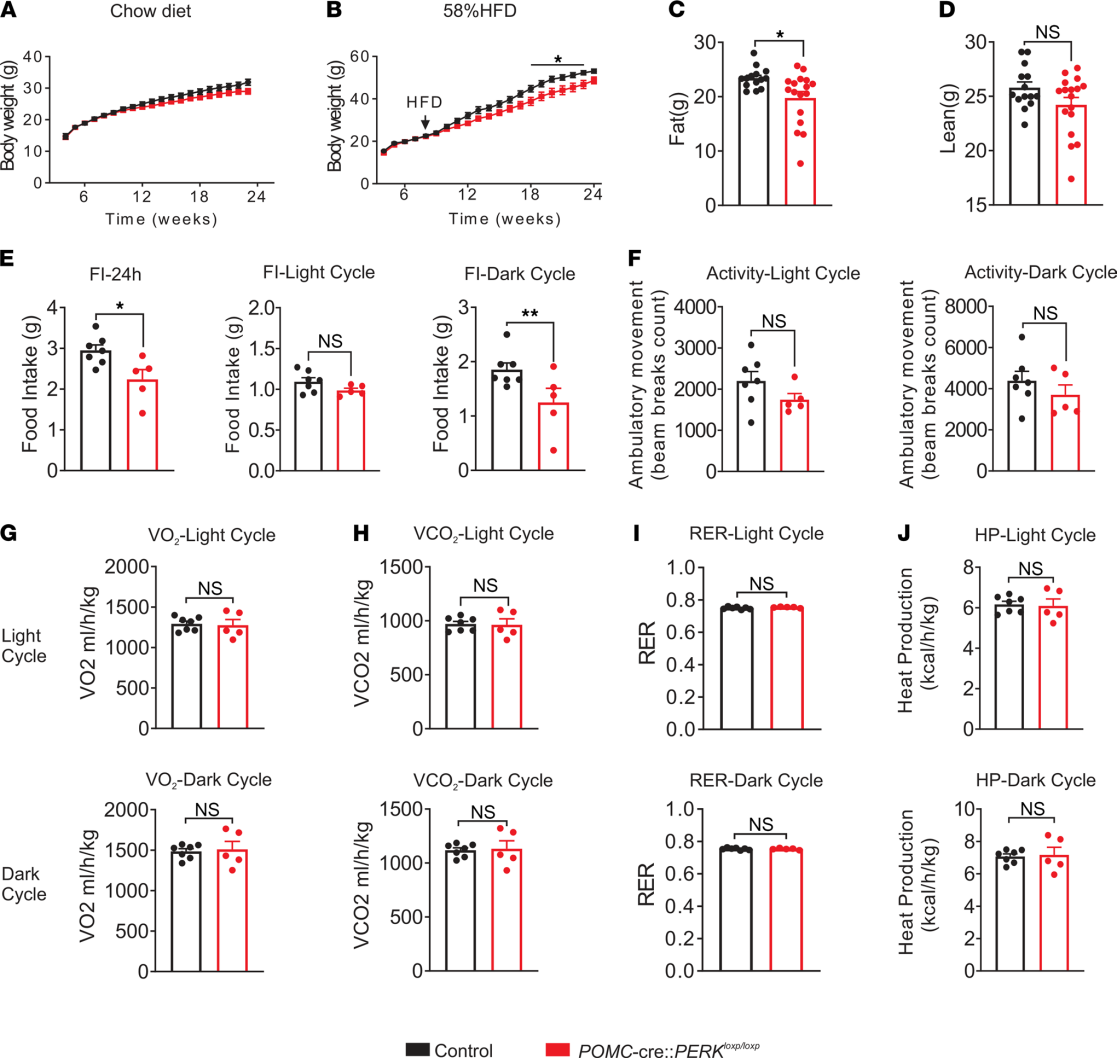
Body weight and metabolic assessment of male POMC-cre:PERK^loxp/loxp^ mice. (**A** and **B**) Body weight curve of male POMC-cre:PERK^loxp/loxp^ and littermate control mice on a chow diet (**A**) or a 58% HFD (**B**). Statistical analyses were performed using 2-way repeated-measures ANOVA, with Bonferroni post hoc analyses applied. (**C** and **D**) Fat mass (**C**) and lean mass (**D**) of male POMC-cre:PERK^loxp/loxp^ and littermate control mice on a 58% HFD at 20 weeks (**A**, *n* = 8–17 per group; **B–D**, *n* = 14–17 per group). (**E**) Male mice with PERK deficiency of POMC neurons displayed decreased food intake in both the 24-hour period and the dark cycle, while the food intake in the light cycle was unaffected. (**F**–**J**) Male POMC-cre:PERK^loxp/loxp^ mice displayed decreased ambulatory activity in both light and dark cycles (**F**), while VO_2_ (**G**), VCO_2_ (**H**), respiratory exchange ratio (RER) (**I**), and heat production (**J**) remained unchanged in both the dark and light cycles. Black bars indicate littermate control mice. Red bars indicate POMC-cre:PERK^loxp/loxp^ mice. (**E–J**) Data are from male mice (*n* = 5–7 per group) and are expressed as mean ± SEM. Unpaired *t* test, *P < 0.05, **P < 0.01.

There was a trend toward leaner body weight in POMC-cre:PERK^loxp/loxp^ mice on a chow diet when compared with that of littermate controls; however, cumulative changes in body weight failed to reach statistical significance (Figure 1A). When fed a high-fat diet (HFD), POMC-cre:PERK^loxp/loxp^ mice displayed an age-dependent lean body weight (Figure 1B). This weight loss was accompanied by a reduction in fat mass [t(29) = 2.642, *P* < 0.05] independent of changes in lean mass (Figure 1, C and D).

To better assess the metabolic parameters, age- and body weight–matched HFD-fed male POMC-cre:PERK^loxp/loxp^ mice and littermate controls were placed in metabolic chambers. POMC-cre:PERK^loxp/loxp^ mice displayed decreased caloric intake, demonstrated by significant decreases of food intake in both the dark cycle [t(10) = 2.327, *P* < 0.05] and the 24-hour monitoring period [t(10) = 2.764, *P* < 0.05, Figure 1E]. No changes were observed in activity, energy expenditure, and heat production (Figure 1, F–J).

Global deletion of PERK leads to diminished postnatal growth and skeletal dysplasia (11, 12). To assess any changes in morphological development, we measured the nose-to-anus length as well as tibial length of age-matched adult POMC-cre:PERK^loxp/loxp^ mice. No significant differences in tibial or linear length were detected in mice deficient for PERK in POMC neurons compared with those in littermate controls (Supplemental Figure 2). These data suggest that POMC-cre:PERK^loxp/loxp^ mice abated the increase in body weight while on HFD when compared with controls through the reduction of food intake, and these phenotypic changes appear independent of any developmental abnormalities, as linear and skeletal growth was similar.

### Deletion of PERK in POMC neurons does not alter glucose metabolism.

**Figure 2:**
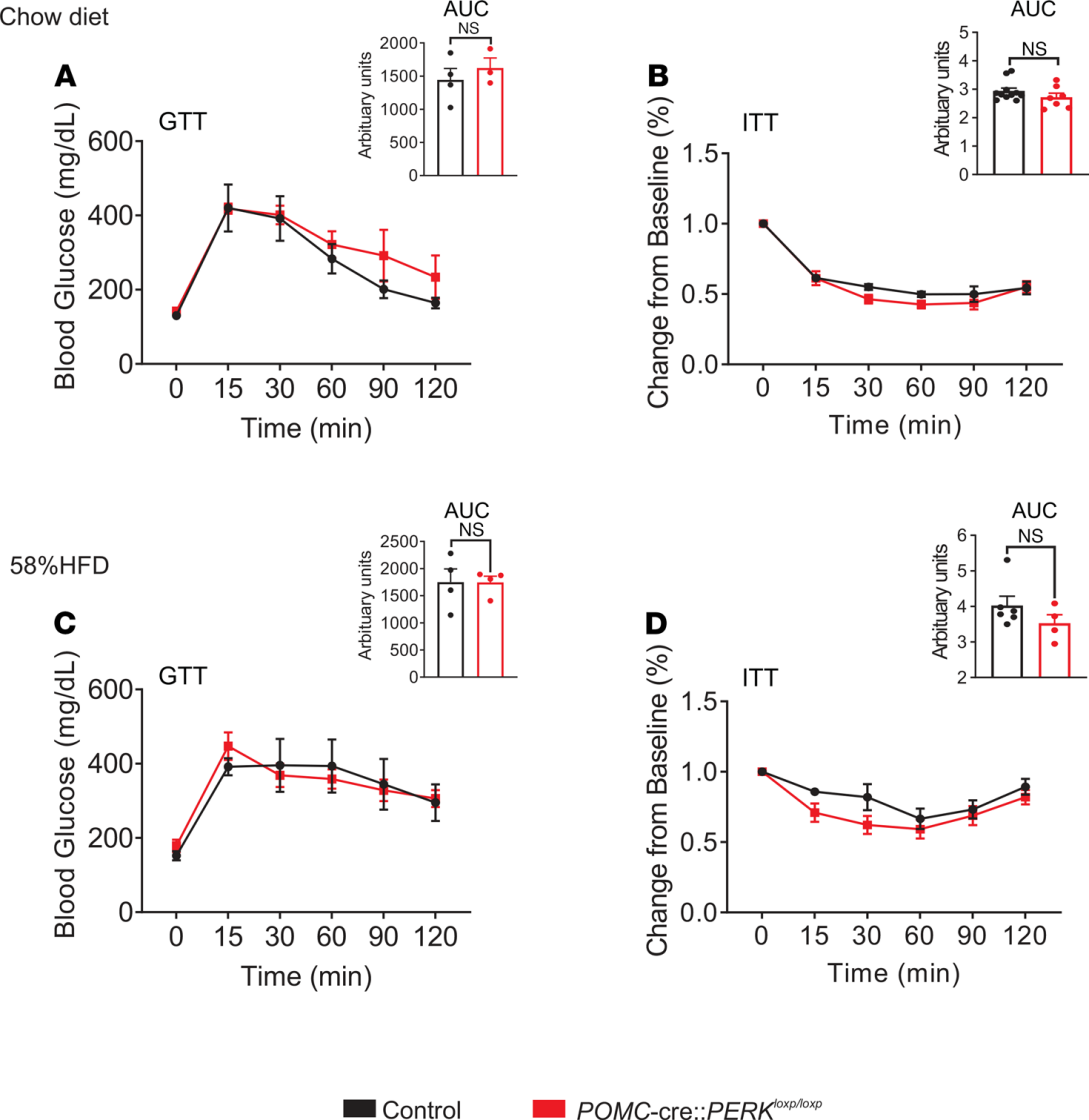
Unaltered glucose metabolism and insulin sensitivity in mice deficient for PERK in POMC neurons. (**A** and **B**) Plots showing the glucose tolerance test (GTT) and insulin tolerance test (ITT) from male POMC-cre:PERK^loxp/loxp^ mice and littermate control mice (*n* = 3–11, per group) fed a chow diet. (**C** and **D**) Plots showing the glucose tolerance test and insulin tolerance test from male POMC-cre:PERK^loxp/loxp^ and littermate control mice (*n* = 4–6, per group) on a 58% HFD. Black bars indicate littermate control mice. Red bars indicate POMC-cre:PERK^loxp/loxp^ mice. Data are from male mice and are expressed as mean ± SEM. AUC, area under the curve. Statistical analyses are performed using 2-way repeated-measures ANOVA, with Bonferroni post hoc analyses applied and area under the curve analyzed by unpaired *t* test.

The effects of PERK are not just limited to decreasing food intake and reduced body weight, as PERK activity modifies peripheral glucose metabolism (18). To further examine the role of PERK in POMC neurons in regulation of glucose metabolism, glucose and insulin tolerance tests (GTTs and ITTs, respectively) were performed on male mice fed either chow or a HFD. Deficiency of PERK in POMC neurons failed to alter both glucose and insulin tolerances in mice on chow or HFD [GTT, chow AUC t(5) = 0.9306, *P* > 0.05; HFD AUC t(6) = 0.01953, *P* > 0.05; ITT, chow AUC t(16) = 0.8750, *P* > 0.05; HFD AUC t(8) = 1.462, *P* > 0.05, Figure 2].

### ER stress markers in the arcuate nuclei of mice deficient for PERK in POMC neurons.

**Figure 3:**
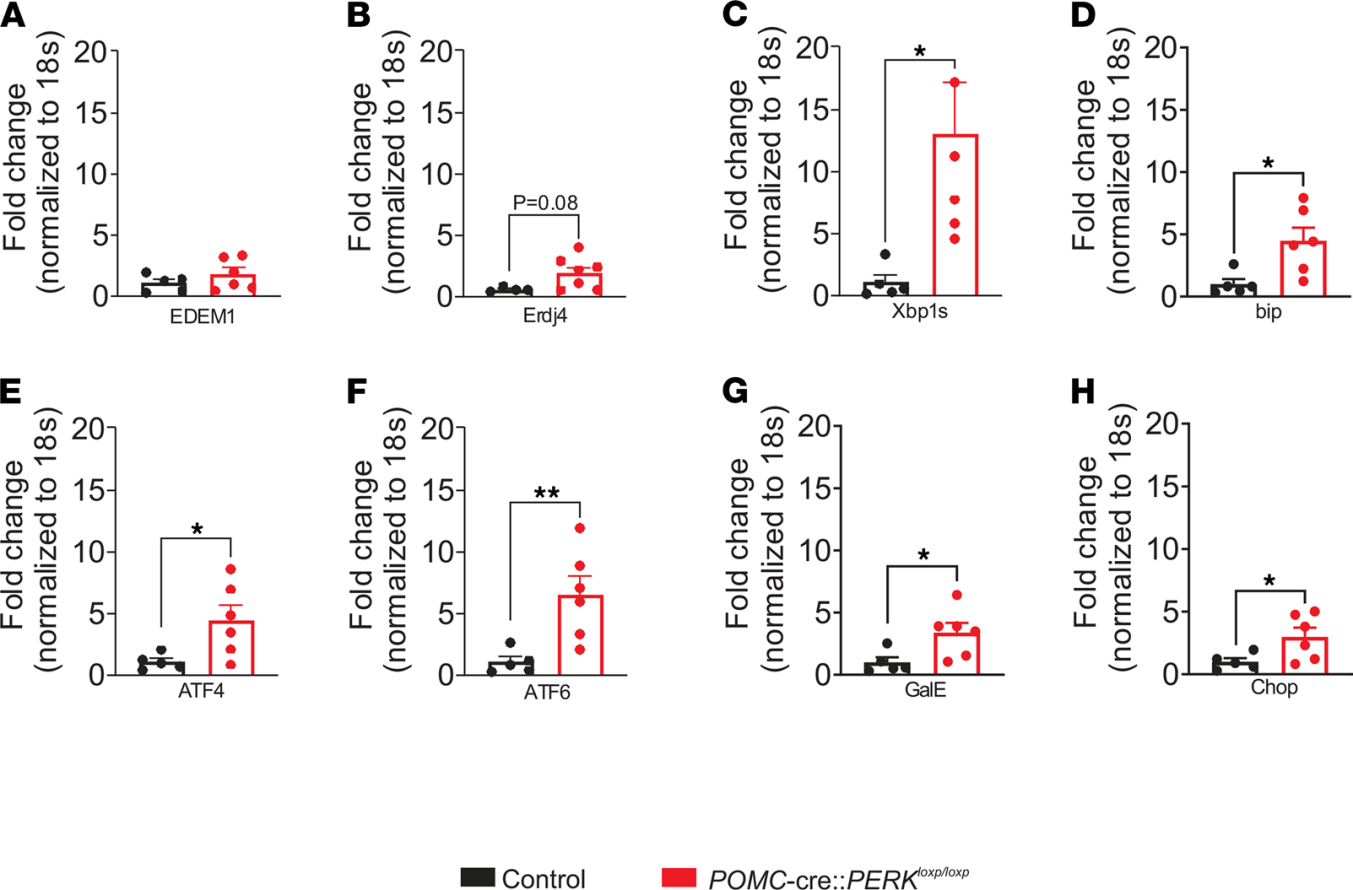
Regulation of ER stress markers in the arcuate nuclei of mice deficient for PERK in POMC neurons. RT-qPCR was performed to examine the relative mRNA expression of (**A**) EDEM1, (**B**) Erdj4, (**C**) Xbp1s, (**D**) bip, (**E**) ATF4, (**F**) ATF6, (**G**) GalE, and (**H**) Chop within the arcuate nuclei from chow-fed POMC-cre:PERK^loxp/loxp^ mice and control littermates (*n* = 4–6, per group). Data are from male mice and are expressed as mean ± SEM. Unpaired *t* test, *P < 0.05; **P < 0.01. Fold change is relative to 18S mRNA.

Given that the ER stress/UPR pathways are commonly interregulated (1, 4, 6, 19–26), we examined whether deficiency of PERK may influence the expression of ER stress/UPR markers. In support of this, we found upregulation of Xbp1s, bip, ATF4, ATF6, GalE, and Chop mRNA within the arcuate nuclei of mice deficient for PERK in POMC neurons [*Xbp1s*, t(9) = 2.598, *P* < 0.05; *bip*, t(9) = 2.838, *P* < 0.05; *ATF4*, t(9) = 2.550, *P* < 0.05; *ATF6*, t(9) = 3.259, *P* < 0.01; *GalE*, t(9) = 2.536, *P* < 0.05; *chop*, t(9) = 2.339, *P* < 0.05, Figure 3]. Erdj4 and EDEM1 also trended toward an increase in expression; however, this trend did not reach statistical significance [*Erdj4*, t(9) = 1.998, *P* = 0.08; *EDEM1*, t(9) = 1.149, *P* > 0.05, Figure 3]. Notably, upregulation of Xbp1s and target genes in the hypothalamus (including POMC neurons) improves energy balance (26). This provides a possible cellular mechanism linking PERK deficiency in POMC neurons to improved metabolism. Moreover, the magnitude of the increased Xbp1s/target gene expression was analogous to the levels observed in the arcuate nucleus in a refed state (26). This supports an expression of ER stress/UPR genes, which mimics a physiological postprandial state in mice deficient for PERK in POMC neurons.

### PERK deletion in POMC neurons blocks the celastrol-induced reduction in both food intake and body weight.

**Figure 4:**
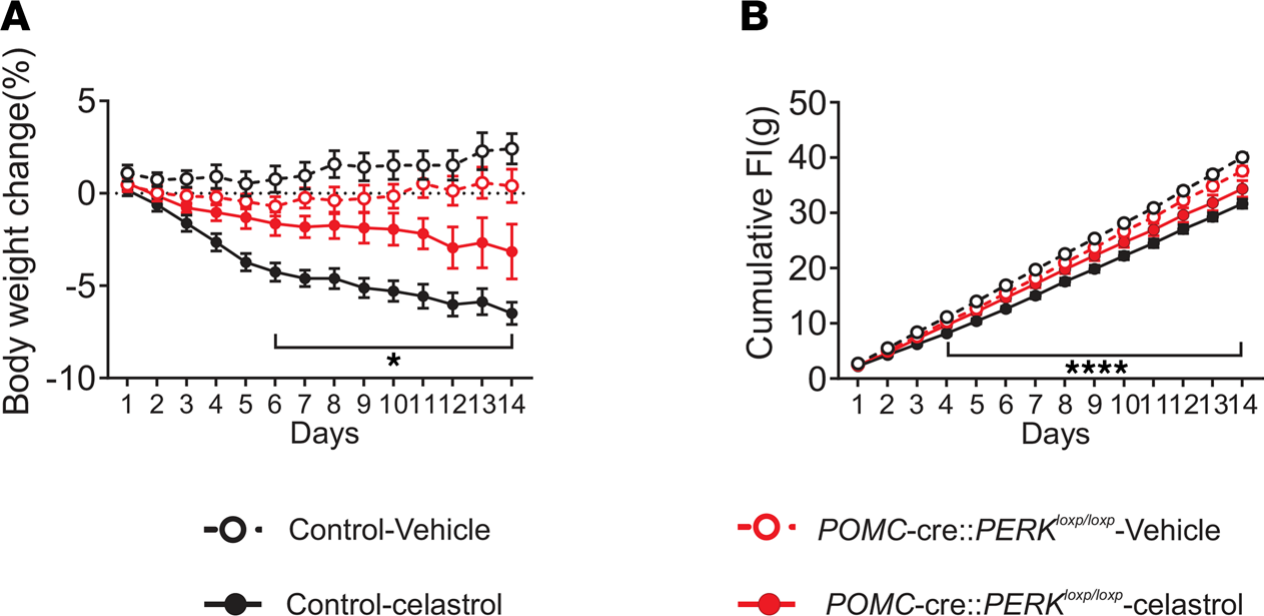
PERK deletion in POMC neurons blunts the effect of celastrol to reduce food intake and body weight. (**A** and **B**) Changes in body weight (**A**) and cumulative food intake (**B**) with celastrol or vehicle i.p. injection daily over 2 weeks in both male POMC-cre:PERK^loxp/loxp^ and littermate control mice (*n* = 7–13, per group). Data are expressed as mean ± SEM. Two-way repeated-measures ANOVA, with Tukey’s post hoc analyses applied, *P < 0.05 for control celastrol against PERK-deficient celastrol; ****P < 0.0001 for control vehicle compared with control celastrol.

Celastrol reduces PERK activity within the hypothalamus (17). Therefore, we hypothesized that PERK in POMC neurons may be required for the beneficial effects of celastrol on food intake and body weight. Body weight and food intake of HFD-fed POMC-cre:PERK^loxp/loxp^ mice and littermate controls were monitored daily while mice received an injection of celastrol for 2 weeks (i.p. injections for 14 days; 100 μg/kg; Q.D.). Similar to previous reports (17, 27), celastrol administration to control mice resulted in a progressive reduction in food intake and body weight (reaching statistical significance after just 6 days of injections, Figure 4). POMC-cre:PERK^loxp/loxp^ mice that received a vehicle injection had a trend toward decreased body weight throughout the experiment in comparison with control vehicle-injected littermates. This is in agreement with the hypophagia and leaner body weight phenotype of these mice. The effect of celastrol in reducing both food intake and body weight was blunted in POMC-cre:PERK^loxp/loxp^ mice (Figure 4). It is noteworthy that celastrol administration in mice deficient for PERK in POMC neurons still resulted in decreased food intake and body weight (reaching significance after 9 days of injection); however, this decrease was not to the magnitude of their control littermates, who also received celastrol after 9 days of celastrol injections, Figure 4). Thus, PERK in POMC neurons is partially required for celastrol to exert its effects on food intake and body weight reduction.

### PERK deletion in POMC neurons abrogates the celastrol-induced increase of leptin sensitivity in vivo.

**Figure 5:**
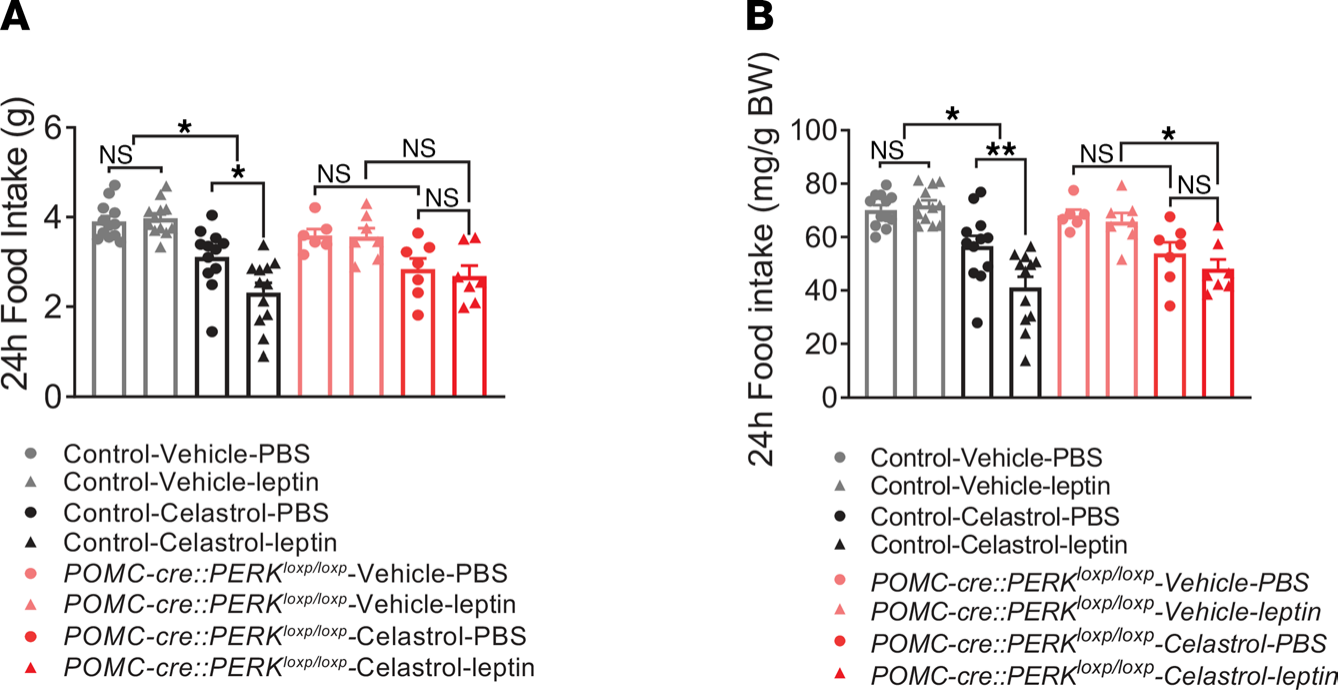
PERK deletion in POMC neurons abrogates the celastrol-induced increase in systemic leptin sensitivity. (**A** and **B**) Twenty-four hour food intake (**A**) and twenty-four hour food intake normalized with body weight (**B**) after leptin (i.p., 5 mg/kg) or PBS injection with/without 2-day pretreatment with celastrol (i.p., 100 μg/kg; *n* = 6–12, per group). Data are from male mice and are expressed as mean ± SEM. Three-way repeated measures ANOVA with Sidak’s post hoc analyses, *P < 0.05; **P < 0.01.

Mice placed on a HFD exhibit decreased sensitivity to leptin (28–34), and celastrol increases leptin sensitivity in DIO mice (17, 35). Similar to that in previous reports, both HFD-fed POMC-cre:PERK^loxp/loxp^ mice and littermate controls exhibited largely decreased responsiveness to leptin treatment (5 mg/kg; i.p., Figure 5). Treatment with celastrol (i.p., 100 μg/kg, 2 days, Q.D.) significantly decreased food intake in wild-type DIO mice (*P* < 0.05); however, no significant change in food intake was observed in HFD-fed POMC-cre:PERK^loxp/loxp^ mice (*P* > 0.05, Figure 5). Moreover, pretreatment with celastrol (i.p., 100 μg/kg, 2 days, Q.D.) improved leptin sensitivity of wild-type DIO mice (Figure 5). However, this effect was absent in mice with POMC-specific PERK deletion (Figure 5).

### Celastrol improves the leptin-induced activation of LepR-expressing POMC neurons from DIO mice in a PERK-dependent manner.

Celastrol has recently emerged as a leptin sensitizer and an ER stress–reducing agent (17, 35). Leptin is an anorectic hormone secreted by adipose tissue and has been shown to regulate food intake and metabolism, at least in part, through activation of arcuate POMC neurons (36). Therefore, we hypothesized that celastrol may decrease body weight and food intake by improving leptin sensitivity of arcuate POMC neurons. In order to investigate this hypothesis, we utilized a transgenic mouse model that labeled POMC neurons with GFP and leptin receptor with tdTomato for electrophysiological studies.

Intrinsic properties of arcuate POMC neurons are dependent upon diet and age (37–39). In particular, a 2-week HFD exposure hyperpolarized and decreased the firing frequency of arcuate POMC neurons (39). Similarly, POMC neurons are inhibited progressively from 2 weeks to 6 months of age (38). In the current study, HFD exposure (8 weeks and 10 months) hyperpolarized arcuate POMC neurons with respect to chow-fed mice, with a decrease in firing frequency observed after HFD feeding for 10 months (Supplemental Figure 3, A and B). The proportion of electrically silent POMC neurons also increased with age and HFD exposure, from 19% for mice fed chow and 17% for mice fed HFD for 8 weeks to 38% at 10 months for mice fed HFD (Supplemental Figure 3C). Interestingly, deletion of PERK in POMC neurons abrogated the age- and HFD-induced suppression of POMC activity, such that POMC neurons from mice fed a HFD for 10 months resembled POMC neurons from mice fed chow for 8 weeks (Supplemental Figure 3, A–C).

**Figure 6:**
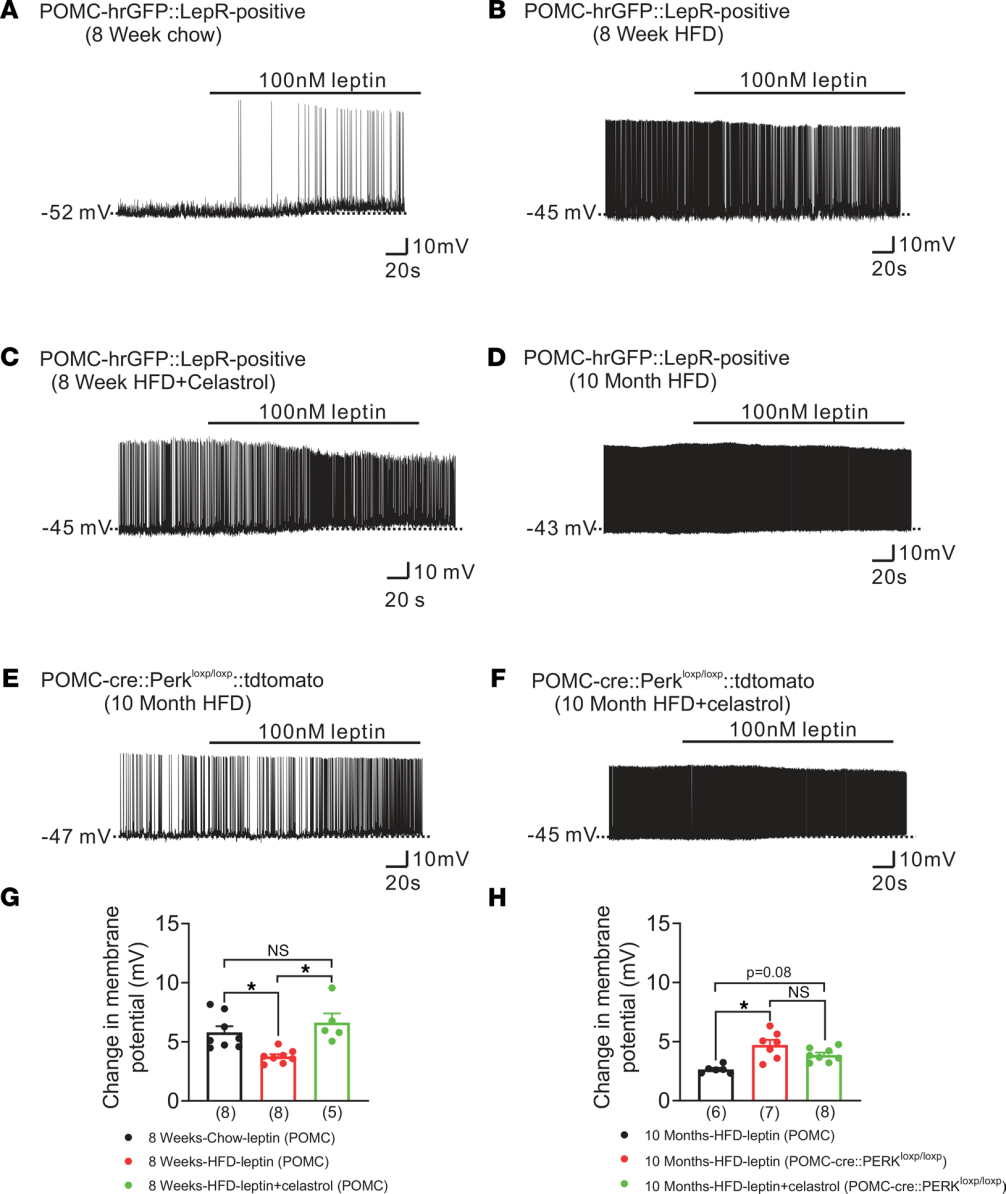
Celastrol-induced restoration of leptin sensitivity in POMC neurons from DIO mice requires PERK. (**A**) A leptin-induced (100 nM) depolarization of LepR-expressing POMC neurons from mice fed a chow diet for 8 weeks. (**B**) Representative leptin-induced (100 nM) depolarization of LepR-expressing POMC neurons from mice fed a HFD for 8 weeks. (**C**) An example of leptin-induced (100 nM) depolarization of LepR-expressing POMC neurons from mice fed a HFD for 8 weeks and treated with celastrol (i.p., 100 μg/kg, Q.D.) for 2 days. (**D**) A leptin-induced (100 nM) depolarization of LepR-expressing POMC neurons from mice fed a HFD for 10 months. (**E**) A leptin-induced (100 nM) depolarization of POMC neurons from POMC-cre:PERK^loxp/loxp^:tdtomato mice fed a HFD for 10 months. (**F**) Representative leptin-induced (100 nM) depolarization of POMC neurons from POMC-cre:PERK^loxp/loxp^:tdtomato mice fed a HFD for 10 months and treated with celastrol (i.p., 100 μg/kg, Q.D.) for 2 days. (**G** and **H**) Histograms summarizing the effect of leptin (100 nM) on the membrane potential of POMC neurons from chow-fed and HFD-fed (8 weeks or 10 months) mice, with/without celastrol treatment. (**G**) The black bar shows the effects of leptin on POMC neurons from mice fed chow for 8 weeks (*n* = 8). The red bar shows the effects of leptin on POMC neurons from mice fed a HFD for 8 weeks (*n* = 8). The green bar shows the effects of leptin on POMC neurons from mice fed a HFD for 8 weeks, which were also injected with celastrol (100 μg/kg for 2 days, Q.D., *n* = 5). (**H**) The black bar shows the effects of leptin on POMC neurons from mice fed a HFD for 10 months (*n* = 6). The red bar shows the effects of leptin on POMC neurons from POMC-cre:PERK^loxp/loxp^:tdtomato mice fed a HFD for 10 months (*n* = 7). The green bar shows the effects of leptin on POMC neurons from POMC-cre:PERK^loxp/loxp^:tdtomato mice fed a HFD for 10 months, which also received an injection of celastrol (i.p., 100 μg/kg for 2 days, *n* = 8). Data are expressed as mean ± SEM. Two-way ANOVA with Tukey’s post hoc analyses, *P < 0.05.

Consistent with that in previous reports (40–45), we found that leptin depolarized leptin receptor–expressing POMC neurons from mice fed a chow diet (change of resting membrane potential, +5.8 ± 0.5 mV, *n* = 8, Figure 6, A and G). POMC neurons from mice fed a HFD for 8 weeks displayed a blunted leptin-induced depolarization when compared with those from chow-fed mice (change of resting membrane potential, +4.0 ± 0.4 mV, *n* = 8, *P* < 0.05, Figure 6, B and G). Injection of celastrol (i.p., 100 μg/kg, for 2 days, Q.D.) in mice that were fed a HFD for 8 weeks restored the leptin-induced depolarization of POMC neurons to that previously observed in chow-fed mice (change of resting membrane potential, +6.7 ± 0.8 mV, *n* = 5, *P* < 0.05, Figure 6, C and G). This effect appeared independent of a direct action of celastrol on cellular properties of POMC neurons, as acute celastrol (10 μM) administration failed to alter the resting membrane potential of arcuate POMC neurons from mice fed either chow (change of resting membrane potential: –0.1 ± 0.2mV, *n* = 18) or HFD (change of resting membrane potential: 0.2 ± 0.2mV, *n* = 14, *P* > 0.05, Supplemental Figure 4, A–C).

To investigate the requirement of PERK for celastrol to improve leptin sensitivity in arcuate POMC neurons, we targeted POMC neurons with and without PERK for electrophysiological recordings (from POMC-cre:tdtomato and POMC-cre:PERK^loxp/loxp^:tdtomato mice, respectively). These mice were fed a HFD for 10 months before we performed patch-clamp recordings. Similar to those from mice fed a HFD for 8 weeks, POMC neurons from mice that were fed a HFD for 10 months displayed a blunted leptin-induced depolarization (change of resting membrane potential, +2.6 ± 0.1 mV, *n* = 6, Figure 6, D and H). POMC neurons deficient for PERK exhibited an enhanced leptin-induced depolarization when compared with those from mice fed a HFD for 10 months (change of resting membrane potential, +5.0 ± 0.4 mV, *n* = 6, *P* < 0.05, Figure 6, E and H). This is notable given that celastrol has been suggested to increase leptin sensitivity via inhibition of PERK activity (17). In support of these data, injection of celastrol (i.p., 100 μg/kg, for 2 days, Q.D.) in mice deficient for PERK in POMC neurons (from POMC-cre:PERK^loxp/loxp^:tdtomato mice) failed to further enhance the leptin-induced depolarization of arcuate POMC neurons (change of resting membrane potential, +3.9 ± 0.2 mV, *n* = 8, Figure 6, F and H).

## Discussion

Selective loss of PERK in POMC neurons decreases food intake, desensitizes mice to DIO independent of changes in energy expenditure and glucose homeostasis. At the cellular level, POMC neurons from POMC-cre:PERK^loxp/loxp^ mice exhibited an enhanced sensitivity to leptin on a HFD and a constitutive increased excitability, likely contributing to improved metabolism. Moreover, the ability of the leptin sensitizer celastrol to sensitize the acute pharmacological effects of leptin on food intake and body weight required, at least in part, PERK in POMC neurons. Celastrol-induced enhancement of acute leptin-induced effects on POMC cellular activity also required PERK in POMC neurons. Finally, decreased expression of PERK in only POMC neurons was sufficient to drive a transcriptional program of ER stress/UPR pathways in the arcuate nucleus analogous to that observed during a fed state. Together, these data support a model in which a fed signal in POMC neurons abrogates HFD-induced obesity (Supplemental Figure 5).

It is of particular interest that decreased PERK expression in *POMC* neurons improves adiposity on a HFD and that impaired PERK activity in POMC neurons seems to be required, at least in part, for the celastrol-induced hypophagia and decreased body weight. This may appear surprising, given that systemic celastrol administration to DIO mice resulted in substantial reduction of food intake and body weight, even in mice with MC4R deficiency, suggesting a melanocortin-independent antiobesity effect of celastrol (27). While MC4R is a major mediator of melanocortin signaling on energy balance and glucose homeostasis, it is also important to note that POMC neurons release neurotransmitters, including glutamate (46). Glutamate via ionotropic receptors has been shown to contribute to energy balance via POMC neurons (46, 47). In particular, deficiency of the vesicular glutamate transporter (*vGlut2*) in POMC neurons results in an age-dependent increase in body weight of male mice on a HFD (46). Moreover, restoration of POMC expression selectively in vGlut2 positive neurons normalized body weight and adiposity (47). These data highlight a parallel role of glutamate in POMC neurons to regulate energy balance. This further suggests a mechanism by which PERK and celastrol may improve metabolism via POMC neurons independent of downstream melanocortin receptors (Supplemental Figure 6). Importantly, it remains unclear if glutamate and melanocortin signaling might work synergistically in celastrol’s activity or if this mechanism may be a compensatory role for glutamate in the absence of melanocortin signaling. Thus, the role of glutamatergic signaling in the PERK pathway and celastrol-induced improvements in energy balance warrants future investigation.

Another salient finding is that deletion of PERK in POMC neurons was only responsible for some of the celastrol-induced, or celastrol and leptin–induced, effect to lower food intake and body weight. This suggests that other sites of action may be required for the beneficial effects of celastrol. These sites can include hypothalamic as well as extrahypothalamic sites of action (48). A prime candidate for celastrol to increase leptin sensitivity is the arcuate agouti-related peptide (AgRP) neuronal population (Supplemental Figure 6). Similar to POMC neurons, arcuate AgRP neurons are leptin responsive and have been shown to be critical regulators of energy balance (49). In particular, Xu and colleagues demonstrated that AgRP neurons might be a primary site of action for leptin to regulate energy balance and glucose homeostasis in the central nervous system (49). However, there might also be a broader role for GABAergic neurons within the arcuate nucleus as primary conduits for mediating leptin action, making AgRP neurons dispensable (50, 51). It should be noted that systemic effects of celastrol might also occur independent of leptin receptor signaling (27). While either scenario does not preclude action within the arcuate nucleus, this casts a wider net for the ability of celastrol to act within specific cell populations or nuclei in regulating energy and glucose homeostasis. Thus, the activity of PERK in POMC neurons appears to mediate part of these effects; however, additional work is warranted to better understand the distributed network of neurons involved in celastrol’s action on metabolism (Supplemental Figure 6).

Eating leads to a decreased motivation for food and lowered glucose production (52–58). Accordingly, stimulation of arcuate POMC neurons suppresses caloric intake, while activation of arcuate NPY/AgRP neurons stimulates feeding (59–61). Leptin, which activates POMC neurons and inhibits NPY/AgRP neurons, improves hepatic glucose metabolism (62–64). In the liver as well as arcuate POMC neurons, refeeding results in a transcriptional program with increased levels of Xbp1s and target genes (26, 65, 66). These analogous effects were observed within the arcuate nuclei of POMC-cre:PERK^loxp/loxp^ mice. This suggests that deletion of PERK alone in POMC neurons drives a similar postprandial transcriptional program, which may contribute to the improvements in systemic metabolism.

While the focus of this study was on arcuate POMC neurons, it is important to note that POMC neurons are not only restricted to the arcuate nucleus. Rather, POMC neurons are also expressed in the hindbrain nucleus tractus solitarius (NTS) (61). NTS POMC neurons have been demonstrated to regulate energy balance; however, the effects appear temporally distinct from arcuate POMC neurons (61). In particular, activation of NTS POMC neurons suppresses acute feeding while activation of arcuate POMC neurons inhibits chronic energy intake (59, 61, 67). Moreover, arcuate POMC neurons (independent of NTS POMC neurons) are sufficient for the anorectic and glucoregulatory effects of agents shown to induce weight-loss and improve glycemic control (68, 69). In the current study, PERK deficiency in POMC neurons leads to chronic decreases in food intake and body weight. While we cannot exclude a possible role for NTS POMC neurons in these changes of metabolism, arcuate POMC neurons may play a more pivotal role in these changes, due to the chronic nature of the observations reported herein.

Finally, mutations in the gene encoding for PERK (*Eif2ak3*) have been associated with WRS in humans (11, 12, 70–73). WRS is a rare genetic disorder that is defined by neonatal/early-onset diabetes mellitus that requires insulin; it is typically associated with skeletal dysplasia and developmental deficiencies (11). Mice with global deletion of PERK exhibit skeletal, pancreatic, and growth defects, which are similar to those seen in human WRS (12, 71, 74–76). Pharmacological impairment of PERK activity within the hypothalamus has been suggested to improve body weight and glucose homeostasis. In the current study, PERK deficiency in POMC neurons decreased body weight largely dependent upon decreased food intake. Importantly, postnatal growth retardation or skeletal deformities were not observed in POMC-cre:PERK^loxp/loxp^ mice compared with their littermates. These data indicate that the improvements in body weight and adiposity of POMC-cre:PERK^loxp/loxp^ mice are independent of growth abnormalities.

### Conclusion.

In summary, the specific deletion of PERK in arcuate POMC neurons attenuates DIO, as both food intake and body weight are decreased. The decreased food intake and lowered body weight observed appears independent of the developmental abnormalities seen in WRS, suggesting that PERK is involved in the regulation of energy balance separate from developmental defects of the disease. Moreover, the pentacyclic triterpenoid celastrol requires PERK in POMC neurons, at least in part, to increase leptin sensitivity and improve energy balance in obese mice. Together, these data highlight the role of PERK in POMC neurons as essential for celastrol’s beneficial effects on leptin sensitivity and energy balance.

## Methods

### Animals.

Male pathogen-free mice were used for all experiments. All mice were housed under standard laboratory conditions (12-hour light/dark cycle; lights on at 7:00 am) in a temperature-controlled environment. Mice were provided a Harlan Teklad 2016 chow diet or high-fat/high-sucrose diet (D12331; Research Diets) and water ad libitum unless otherwise noted. To yield PERK deletion in POMC neurons, POMC-cre mice were crossed with PERK^loxp/loxp^ mice to generate POMC-cre:PERK^loxp/loxp^ mice. To identify POMC neurons with or without leptin receptors, we generated POMC-hrGFP:LepR-cre:tdtomato mice and targeted POMC neurons anatomically restricted to the arcuate nucleus of the hypothalamus as previously described (77).

### Body weight and body composition measurement.

Body weight was measured weekly up to 23–25 weeks, and body composition was measured by using nuclear magnetic resonance (Bruker minispec; Bruker Corporation). Chow diets were replaced by HFD at 8 weeks.

### Auxological data.

Mice were sacrificed at 38 weeks, and tibiae were harvested. Tibia length was measured using an electronic digital caliper (500-196-30; Mitutoyo Corp.) after dissection of the surrounding tissues and careful disarticulation of the bones. The right tibia length was considered as the maximal distance between proximal condyles and malleolus. To determine the effects of the loss of PERK expression specific to POMC neurons on body length, nose-to-anal distance was measured using an electronic digital caliper (500-196-30; Mitutoyo Corp.).

### Metabolic chambers.

Experiments were performed in a temperature-controlled room containing 36 TSE Systems metabolic cages maintained by personnel at The University of Texas Southwestern Animal Resource Center. One week before the study, mice were singly housed to acclimate to new housing. Three days before the study, mice were transported to the room containing the metabolic cages to acclimate to a new environment. A high-fat/high-sucrose diet, if applicable, was also introduced at the beginning of this acclimation period. After 3 days of acclimation, cages were connected to the TSE Systems metabolic cages for a total of 5 days. Days 2–4 were used for data analyses. Mice that incurred a 10% body weight loss during the acclimation period for metabolic cage studies (described below) were not used.

### GTTs.

After a 4-hour fast, 10- to 14-week-old male mice received i.p. injections of 1.5 g/kg d-glucose. Blood glucose was measured from tail blood using a glucometer at serial time points as indicated in the figures.

### ITTs.

After a 4-hour fast to empty the stomach, 10- to 14-week-old male mice received i.p. injections of insulin (1.2 units/kg). Blood glucose was measured from tail blood as described previously (19, 78).

### Analysis of gene expression by quantitative PCR.

Total RNA was extracted from tissues with RNA STAT-60 reagent (Tel Test Inc.) according to the manufacturer’s instructions. Total RNA (1 mg) was converted into first-strand cDNA with oligo(dT) primers per the manufacturer’s instructions (Applied Biosystems). PCR was performed in a CFX96 Touch Real-Time Q-PCR system (Bio-Rad) with specific primers (Supplemental Table 1) and SYBR Green PCR Master Mix (Roche Life Science). The relative abundance of mRNAs was standardized with 18S mRNA as the invariant control.

### Blood collection.

Blood samples were collected via tail using heparinized capillary tubes. The blood samples were immediately centrifuged at 4°C and 1500*g* for 15 minutes, and plasma were stored at 80°C for further biochemical analyses.

### Slice preparation.

Brain slices were prepared from male mice as previously described (19, 40, 78–81). Briefly, male mice were deeply anesthetized with i.p. injections of 7% chloral hydrate and transcardially perfused with a modified ice-cold artificial CSF (ACSF) (described below) (80). The mice were then decapitated, and the entire brain was removed and immediately submerged in ice-cold, carbogen-saturated (95% O_2_ and 5% CO_2_) ACSF (126 mM NaCl, 2.8 mM KCl, 1.2 mM MgCl_2_, 2.5 mM CaCl_2_, 1.25 mM NaH_2_PO_4_, 26 mM NaHCO_3_, and 5 mM glucose).

Coronal sections (250 mm) were cut with a Leica VT1000S Vibratome and then incubated in oxygenated ACSF at room temperature for at least 1 hour before recording. The slices were bathed in oxygenated ACSF (32°C–34°C) at a flow rate of approximately 2 ml/min.

### Whole-cell recording.

The pipette solution was modified to include an intracellular dye (Alexa Fluor 350 hydrazide dye (Thermo Fisher Scientific) for whole-cell recording: 120 mM K-gluconate, 10 mM KCl, 10 mM HEPES, 5 mM EGTA, 1 mM CaCl_2_, 1 mM MgCl_2_, 2 mM MgATP, and 0.03 mM Alexa Fluor 350 hydrazide dye (pH 7.3). Epifluorescence was briefly used to target fluorescent cells, at which time the light source was switched to infrared differential interference contrast imaging to obtain the whole-cell recording (Zeiss Axioskop FS2 Plus equipped with a fixed stage and a QuantEM:512SC electron-multiplying charge-coupled device camera). Electrophysiological signals were recorded using an Axopatch 700B amplifier (Molecular Devices), low-pass filtered at 2–5 kHz, and analyzed offline on a PC with pCLAMP programs (Molecular Devices). Membrane potential and firing rate were measured by whole-cell current clamp recordings from POMC in brain slices. Recording electrodes had resistances of 2.5–5 MΩ when filled with the K-gluconate internal solution.

Solutions containing drugs were typically perfused for 5 minutes. A drug effect was required to be associated temporally with compound application, and the response had to be stable within a few minutes. A change in membrane potential was required to be at least 2 mV in amplitude, the onset was required to be associated temporally with the peptide application (i.e., usually beginning at about 1–2 minutes after changing solutions, the time it took for compound to arrive at the recording chamber), and the response had to be saturated and stable within a few minutes (i.e., did not continually change). The value of the membrane potential was measured at a specific time after compound application (i.e., 3–4 min after the compound arrived in the chamber and no continual changes).

### Drugs.

Leptin (100 nM and i.p. 5 mg/kg, dissolved in Dulbecco’s PBS from Gibco) was provided by A.F. Parlow (Torrance, California, USA), through the National Hormone and Peptide Program. Celastrol (10 μM and 100 μg/kg, dissolved in DMSO from MilliporeSigma) was from Tocris. The final concentration of dimethyl sulfoxide applied to the slice was <0.1%.

### Animal studies.

For the leptin and celastrol daily injection study, age-matched male control and POMC-cre:PERK^loxp/loxp^ mice were single housed and fed with chow or HFD diet. Food intake (24 h) and body weight were measured at 9:00 am for 5 consecutive days as a baseline. Starting from day 6, celastrol (100 μg/kg), vehicle (DMSO and sterile saline, 10 ml/kg), and leptin (5 mg/kg) were administered i.p. in a counterbalanced manner to both controls and POMC-cre:PERK^loxp/loxp^ mice at 9:00 am. The final concentration of dimethyl sulfoxide applied to the slice was ≤0.3%. Body weight and food intake were measured daily.

### Statistics.

Results are reported as the mean ± SEM unless indicated otherwise. Significance was set at *P* < 0.05 for all statistical measures. All data were evaluated using a 2-tailed Student’s *t* test or 2- or 3-way ANOVA with post hoc analyses where applicable. All graphs were made using Graph Pad Prism 8.0 software. All figures were created using CorelDraw C8 (64 Bit).

### Study approval.

All experiments were performed in accordance with the guidelines established by the *Guide for the Care and Use of Laboratory Animals* (National Academies Press, 2011) and were approved by The University of Texas Institutional Animal Care and Use Committee.

## Author contributions

KWW conceived and designed the study. ZH, LL, and YD are co–first authors. Author order was determined with the following considerations. ZH and LL initialized the study, analyzed data, organized figures, and wrote the manuscript. ZH managed major parts of the electrophysiological experiments and food intake collection. LL managed metabolic phenotyping of the mice. YD also contributed to electrophysiological and food intake experiments and analyzed and organized data and the manuscript at later stages. ZH, YD, JC, and DC designed and performed electrophysiological experiments, analyzed data, and wrote the manuscript. LL, YD, SA, DC, AK, BW, ESH, TY, YH, and BC designed and performed all experiments, except electrophysiological experiments; analyzed the data; and wrote the manuscript. JO assisted with experiments. YG, LH, and KWW designed experiments and edited the manuscript.

## Supplementary Material

Supplemental data

## Version 1 09/22/2021

Electronic publication
