# Supplementary material for: PERK in POMC neurons connects celastrol with metabolism
Source: JCI Insight. 2021 Sep 22;6(18):e145306. doi: 10.1172/jci.insight.145306 (PMC8492333; doi:10.1172/jci.insight.145306)
Supplement: Supplemental data [file jciinsight-6-145306-s287.pdf]

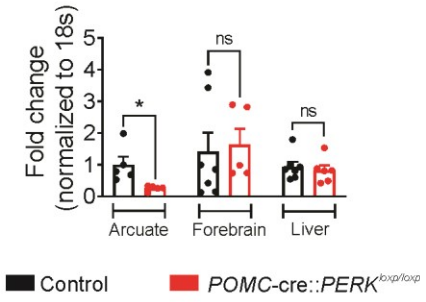

Supplement Figure 1

Figure S1: qPCR validation of PERK specific deletion in arcuate POMC neurons

(A) qPCR detection of PERK mRNA expression in the arcuate nucleus of hypothalamus, forebrain, and liver, of mice with POMC neuron-specific deficiency of PERK (POMC-cre::PERK$^{loxp/loxp}$) and littermate controls (POMC-cre::PERK$^{+/+}$). Black bars: littermate control mice. Red bars: POMC-cre::PERK$^{loxp/loxp}$ mice. Data are from male mice (n=5-7, per group) analyzed using unpaired t-test and are expressed as mean ± SEM; *$p < 0.05$.

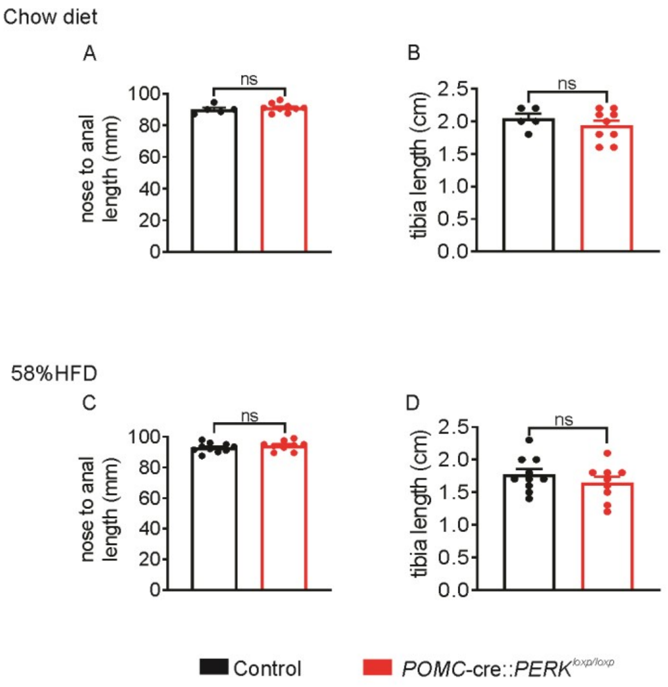

Supplement Figure 2

Figure S2: Body length of male POMC-cre::PERK$^{loxp/loxp}$ mice

(A) Body length of male POMC-cre::PERK$^{loxp/loxp}$ mice on a chow diet at 48 weeks. (B) Tibia length of male POMC-cre::PERK$^{loxp/loxp}$ mice on a chow diet at 48 weeks. (C) Body length of male POMC-cre::PERK$^{loxp/loxp}$ mice on a 58% HFD at 48 weeks. (D) Tibia length of male POMC-cre::PERK$^{loxp/loxp}$ mice on a 58% HFD at 48 weeks (Black bars: WT mice. Red bars: POMC-cre::PERK$^{loxp/loxp}$ mice, n = 5-10, per group). Data are from male mice are expressed as mean ± SEM. Unpaired t-test.

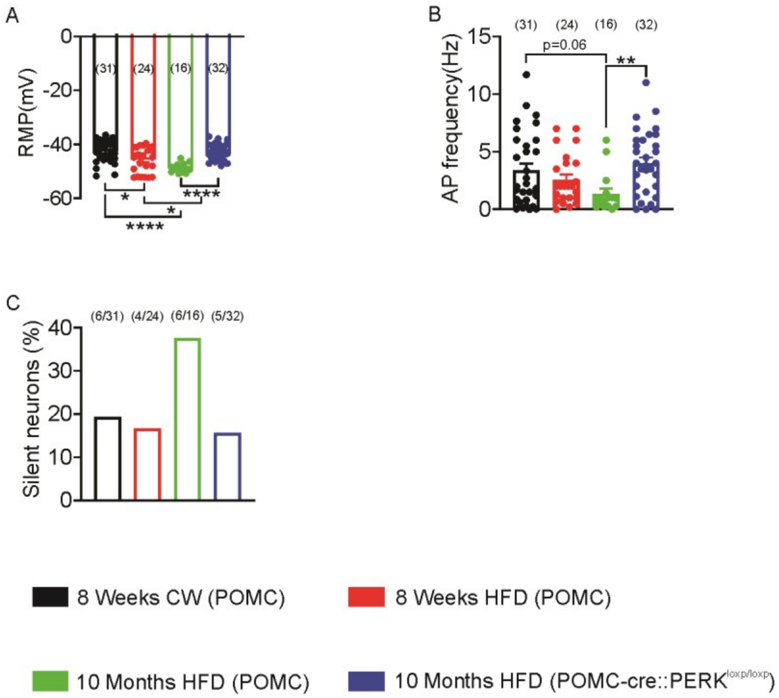

Supplement Figure 3

Figure S3: PERK deletion in POMC neurons depolarized resting membrane potential and activated POMC cells from mice on HFD

Histogram shows the resting membrane potential (A), action potential frequency (B) and percentage silent neurons; F < 0.5 Hz (C) of POMC neurons from POMC-cre::PERK$^{loxp/loxp}$::tdtomato mice and LepR-expressing POMC neurons control mice (Black: LepR-expressing POMC neurons from control mice fed chow diet for 8 weeks, n=31, Red: LepR-expressing POMC neurons from control mice fed HFD for 8 weeks, n=24, Green: LepR-expressing POMC neurons from mice fed HFD for 10 months, n=16, Blue: POMC-

cre::$PERK^{loxp/loxp}$::tdtomato mice fed HFD for 10 months, n=32). Data are expressed as mean ± SEM, Unpaired t-test, **$p<0.01$; ****$p<0.0001$.

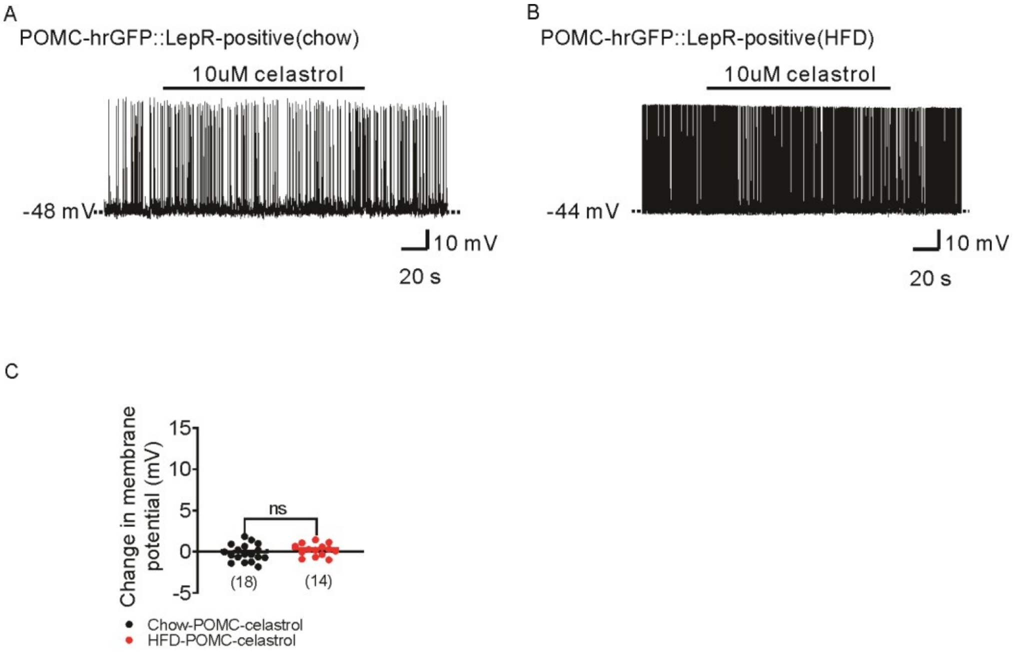

Supplement Figure 4

Figure S4: Acute Celastrol administration fails to alter POMC activity.

(A and B) Electrophysiological recordings demonstrate acute Celastrol (10uM) application fails to alter the resting membrane potential of LepR-expressing POMC neurons from either chow diet (A) or HFD (B) fed mice. (C) Histogram summarizing the acute effect of Celastrol (10uM) on the membrane potential of LepR-expressing POMC neurons from either chow or HFD fed mice (black dots: Celastrol application on LepR-expressing POMC neurons from chow fed mice, n=18; red dots: Celastrol application on LepR-expressing POMC neurons from HFD fed mice, n=14). Data are from male mice expressed as mean ± SEM. Unpaired t-test.

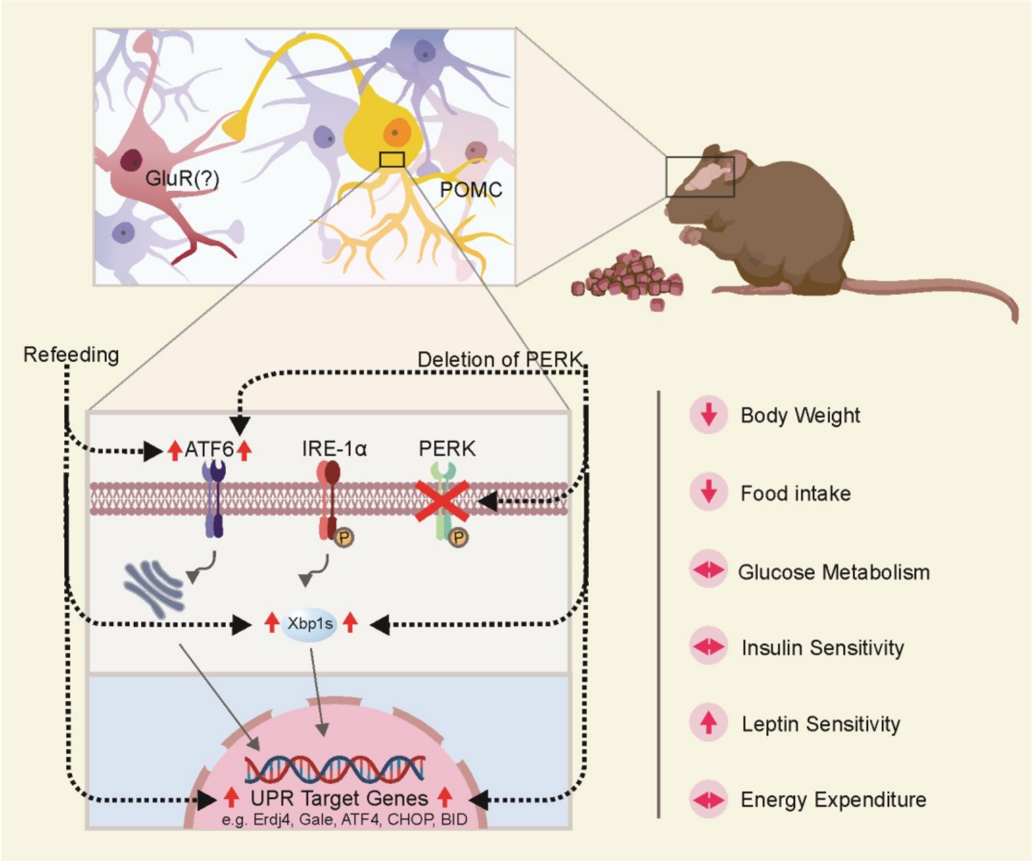

Supplement Figure 5

Figure S5: Physiological requirement of PERK in POMC neurons

Celastrol selectively inhibits the phosphorylation of PERK within the hypothalamus independent of other ER stress/UPR pathways (17). In the current study, we found that selective deficiency of PERK within POMC neurons, alone, mimics this effect of Celastrol. In particular, mice deficient for PERK in POMC neurons exhibited a lowered body weight largely dependent upon decreased food intake and increased systemic leptin sensitivity. Deletion of PERK from POMC neurons also resulted in a transcriptional program that was reminiscent of a post-prandial state, which may also contribute to the improvements in metabolism.

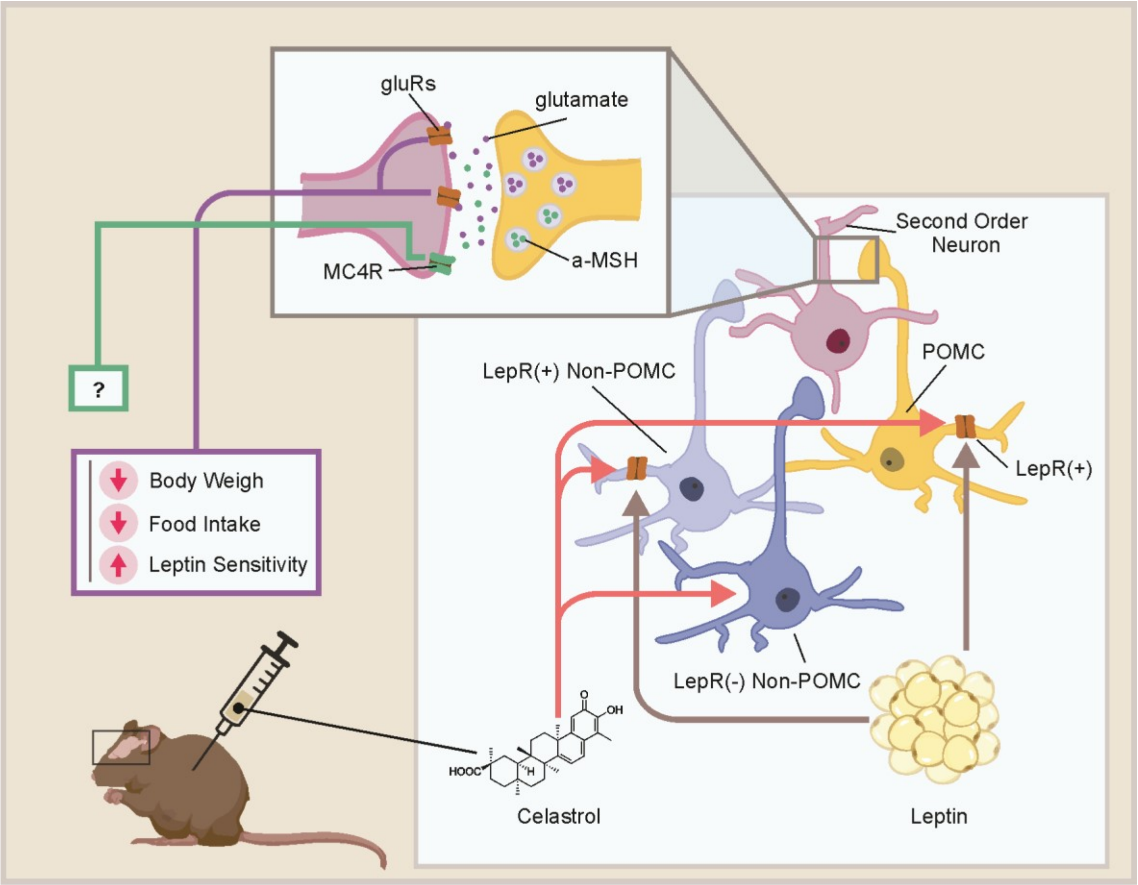

Supplement Figure 6

Figure S6: Pharmacological requirement of PERK in POMC neurons

In the current study, we found that Celastrol required PERK in POMC neurons, at least in part to mediate its beneficial effects on metabolism. PERK in POMC neurons was also required for the Celastrol-induced improvements of leptin sensitivity, both at the cellular and systems level. This highlights an important role of POMC neurons, mediating certain beneficial effects of Celastrol. Interestingly, the Celastrol-induced improvements in energy balance occur independent of MC4R and possibly LEPR signaling (33). Although Celastrol may work independent of MC4R receptors (33), these data cannot exclude the possibility of other receptors, such as receptors

for glutamate (gluRs), in mediating these observed effects downstream of POMC neurons. It is important to note that these data also do not preclude the possibility of other cell populations in this response, as PERK in POMC was partially required for these effects. Thus, based on previous and current experimental results we provide the current model for Celastrol action via PERK activity.

Supplemental Table 1: Primer sequences used for real-time quantitative polymerase chain reaction

Gene	Primer sequence	Ascension #
PEPCK - phosphoenolpyruvate carboxykinase 1 (Pck1)	5'TGACAACTGTTGGCTGGCTC<br>5'GACATACATGGTGCGGCCTT	NM_011044
G6P - Glucose-6- phosphatase (G6pc)	5'GGCGCAGCAGGTGTATACTA<br>5'ATGCCTGACAAGACTCCAGC	NM_008061
HNF4α - Hepatic nuclear factor 4, alpha (Hnf4a)	5'GCATGGATATGGCCGACTAC<br>5'TGTGGTTCTTCCTCACGCTC	NM_008261
Pyruvate carboxylase (Pcx)	5'GTGAGATTGCCATCCGAGTG<br>5'TCTGCTCGCTCTGAGAGGAA	NM_008797
Fox01- Forkhead box protein 01	5'GTGAACACCATGC<br>5'CACAGTCCAAGCG	NM_019739
Eif2aK3 - Eukaryotic Translation Initiation Factor 2AK3, PKR-like endoplasmic reticulum (ER) kinase (PERK)	5'TGGAATGACATGAAGTACTCAGG ATAC<br>5'CCACCACGACCCATGCA	NM_010121
Xbp1 - X-box binding protein 1	5'ACATCTTCCCATGGACTCTG<br>5'TAGGTCCTTCTGGGTAGACC	NM_013842
s-Xbp1 – spliced- X-box binding protein 1	5′GGTCTGCTGAGTCCGCAGCAGG<br>5′GAAAGGGAGGCTGGTAAGGAAC	NM_0133842

Erdj4 - heat shock protein family (Hsp40) member B9 (Dnajb9)	5’CTTAGGTGTGCCAAAGTCTGC<br>5’GGCATCCGAGAGTGTTTCATA	NM_013760
GalE - UDP-galactose-4-epimerase	5’CCATAACGCCATT<br>5’TCCAGAGGCTTCT	NM_001356493.1
Atf4 - activating transcription factor 4	5′CATGCCAGATGAGCTCTTGA<br>5′GCCAATTGGGTTCACTGTCT	NM_009716.2
Atf6 - activating transcription factor 6	5'CAGAAGTATGGGTTCGGATATCG<br>5'TGGCCTCCAGTCCTAGCAT	NM_001081304
Chops - CCAAT-enhancer-binding protein homologous protein, DNA-damage inducible transcript 3 (Ddit3)	5’ATATCTCATCCCCAGGAAACG<br>5’TCTTCCTTGCTCTTCCTCCTC	NM_007837
Bip – Binding immunoglobulin Protein, heat shock protein 5 (Hspa5)	5’GCTTCCGATAATCAGCCAACTG<br>5’GATTGTCTTTTGTTAGGGGTCG	NM_022310
EDEM1 - ER degradation enhancer, mannosidase alpha-like 1	5′TCTCTCCTGGTGGAATTTGG<br>5′AATGGCCTGTCTGGATGTTC	NM_138677

18s - 18S ribosomal RNA	5' ACCGCAGCTAGGAATAATGGA<br>5' GCCTCAGTTCCGAAAACCA	X00686, M10098

References Cited:

1. Ron D, and Walter P. Signal integration in the endoplasmic reticulum unfolded protein response. *Nat Rev Mol Cell Biol.* 2007;8(7):519-29.
2. Ma Y, and Hendershot LM. The unfolding tale of the unfolded protein response. *Cell.* 2001;107(7):827-30.
3. Schroder M, and Kaufman RJ. ER stress and the unfolded protein response. *Mutation research.* 2005;569(1-2):29-63.
4. Hotamisligil GS. Endoplasmic reticulum stress and the inflammatory basis of metabolic disease. *Cell.* 2010;140(6):900-17.
5. Wang M, and Kaufman RJ. Protein misfolding in the endoplasmic reticulum as a conduit to human disease. *Nature.* 2016;529(7586):326-35.
6. Ozcan U, Cao Q, Yilmaz E, Lee AH, Iwakoshi NN, Ozdelen E, et al. Endoplasmic reticulum stress links obesity, insulin action, and type 2 diabetes. *Science.* 2004;306(5695):457-61.
7. Ghemrawi R, Battaglia-Hsu SF, and Arnold C. Endoplasmic Reticulum Stress in Metabolic Disorders. *Cells.* 2018;7(6).
8. Araki E, Oyadomari S, and Mori M. Endoplasmic reticulum stress and diabetes mellitus. *Internal medicine.* 2003;42(1):7-14.
9. Eizirik DL, Cardozo AK, and Cnop M. The role for endoplasmic reticulum stress in diabetes mellitus. *Endocr Rev.* 2008;29(1):42-61.
10. Koumenis C, Naczki C, Koritzinsky M, Rastani S, Diehl A, Sonenberg N, et al. Regulation of protein synthesis by hypoxia via activation of the endoplasmic reticulum kinase PERK and phosphorylation of the translation initiation factor eIF2alpha. *Mol Cell Biol.* 2002;22(21):7405-16.
11. Julier C, and Nicolino M. Wolcott-Rallison syndrome. *Orphanet J Rare Dis.* 2010;5:29.
12. Zhang P, McGrath B, Li S, Frank A, Zambito F, Reinert J, et al. The PERK eukaryotic initiation factor 2 alpha kinase is required for the development of the skeletal system, postnatal growth, and the function and viability of the pancreas. *Mol Cell Biol.* 2002;22(11):3864-74.
13. Li Y, Iida K, O'Neil J, Zhang P, Li S, Frank A, et al. PERK eIF2alpha kinase regulates neonatal growth by controlling the expression of circulating insulin-like growth factor-I derived from the liver. *Endocrinology.* 2003;144(8):3505-13.
14. Kong FJ, Ma LL, Guo JJ, Xu LH, Li Y, and Qu S. Endoplasmic reticulum stress/autophagy pathway is involved in diabetes-induced neuronal apoptosis and cognitive decline in mice. *Clin Sci (Lond).* 2018;132(1):111-25.
15. Yi S, Chen K, Zhang L, Shi W, Zhang Y, Niu S, et al. Endoplasmic Reticulum Stress Is Involved in Stress-Induced Hypothalamic Neuronal Injury in Rats via the PERK-ATF4-CHOP and IRE1-ASK1-JNK Pathways. *Front Cell Neurosci.* 2019;13:190.
16. Gao Y, Sartori DJ, Li C, Yu QC, Kushner JA, Simon MC, et al. PERK is required in the adult pancreas and is essential for maintenance of glucose homeostasis. *Mol Cell Biol.* 2012;32(24):5129-39.
17. Liu J, Lee J, Salazar Hernandez MA, Mazitschek R, and Ozcan U. Treatment of obesity with celastrol. *Cell.* 2015;161(5):999-1011.
18. Sohn JW, Xu Y, Jones JE, Wickman K, Williams KW, and Elmquist JK. Serotonin 2C receptor activates a distinct population of arcuate pro-opiomelanocortin neurons via TRPC channels. *Neuron.* 2011;71(3):488-97.
19. Yao T, Deng Z, Gao Y, Sun J, Kong X, Huang Y, et al. Ire1alpha in Pomc Neurons Is Required for Thermogenesis and Glycemia. *Diabetes.* 2017;66(3):663-73.

20. Huang Y, He Z, Gao Y, Lieu L, Yao T, Sun J, et al. Phosphoinositide 3-Kinase Is Integral for the Acute Activity of Leptin and Insulin in Male Arcuate NPY/AgRP Neurons. *J Endocr Soc.* 2018;2(6):518-32.
21. He Z, Gao Y, Alhadeff AL, Castorena CM, Huang Y, Lieu L, et al. Cellular and synaptic reorganization of arcuate NPY/AgRP and POMC neurons after exercise. *Mol Metab.* 2018;18:107-19.
22. He Z, Gao Y, Lieu L, Afrin S, Cao J, Michael NJ, et al. Direct and indirect effects of liraglutide on hypothalamic POMC and NPY/AgRP neurons - Implications for energy balance and glucose control. *Mol Metab.* 2019;28:120-34.
23. Sun J, Gao Y, Yao T, Huang Y, He Z, Kong X, et al. Adiponectin potentiates the acute effects of leptin in arcuate Pomc neurons. *Mol Metab.* 2016;5(10):882-91.
24. Gao Y, Yao T, Deng Z, Sohn JW, Sun J, Huang Y, et al. TrpC5 Mediates Acute Leptin and Serotonin Effects via Pomc Neurons. *Cell Rep.* 2017;18(3):583-92.
25. Birkenfeld AL, Lee HY, Majumdar S, Jurczak MJ, Camporez JP, Jornayvaz FR, et al. Influence of the hepatic eukaryotic initiation factor 2alpha (eIF2alpha) endoplasmic reticulum (ER) stress response pathway on insulin-mediated ER stress and hepatic and peripheral glucose metabolism. *J Biol Chem.* 2011;286(42):36163-70.
26. Cnop M, Toivonen S, Igoillo-Esteve M, and Salpea P. Endoplasmic reticulum stress and eIF2alpha phosphorylation: The Achilles heel of pancreatic beta cells. *Mol Metab.* 2017;6(9):1024-39.
27. Nakamura T, Furuhashi M, Li P, Cao H, Tuncman G, Sonenberg N, et al. Double-stranded RNA-dependent protein kinase links pathogen sensing with stress and metabolic homeostasis. *Cell.* 2010;140(3):338-48.
28. Cullinan SB, and Diehl JA. Coordination of ER and oxidative stress signaling: the PERK/Nrf2 signaling pathway. *The international journal of biochemistry & cell biology.* 2006;38(3):317-32.
29. Harding HP, Zhang Y, and Ron D. Protein translation and folding are coupled by an endoplasmic-reticulum-resident kinase. *Nature.* 1999;397(6716):271-4.
30. Shi Y, Vattem KM, Sood R, An J, Liang J, Stramm L, et al. Identification and characterization of pancreatic eukaryotic initiation factor 2 alpha-subunit kinase, PEK, involved in translational control. *Mol Cell Biol.* 1998;18(12):7499-509.
31. Harding HP, Zhang Y, Bertolotti A, Zeng H, and Ron D. Perk is essential for translational regulation and cell survival during the unfolded protein response. *Mol Cell.* 2000;5(5):897-904.
32. Williams KW, Liu T, Kong X, Fukuda M, Deng Y, Berglund ED, et al. Xbp1s in Pomc neurons connects ER stress with energy balance and glucose homeostasis. *Cell Metab.* 2014;20(3):471-82.
33. Saito K, Davis KC, Morgan DA, Toth BA, Jiang J, Singh U, et al. Celastrol Reduces Obesity in MC4R Deficiency and Stimulates Sympathetic Nerve Activity Affecting Metabolic and Cardiovascular Functions. *Diabetes.* 2019;68(6):1210-20.
34. de Luca C, Kowalski TJ, Zhang Y, Elmquist JK, Lee C, Kilimann MW, et al. Complete rescue of obesity, diabetes, and infertility in db/db mice by neuron-specific LEPR-B transgenes. *J Clin Invest.* 2005;115(12):3484-93.
35. Chen H, Charlat O, Tartaglia LA, Woolf EA, Weng X, Ellis SJ, et al. Evidence that the diabetes gene encodes the leptin receptor: identification of a mutation in the leptin receptor gene in db/db mice. *Cell.* 1996;84(3):491-5.
36. Halaas JL, Gajiwala KS, Maffei M, Cohen SL, Chait BT, Rabinowitz D, et al. Weight-reducing effects of the plasma protein encoded by the obese gene. *Science.* 1995;269(5223):543-6.
37. El-Haschimi K, Pierroz DD, Hileman SM, Bjorbaek C, and Flier JS. Two defects contribute to hypothalamic leptin resistance in mice with diet-induced obesity. *J Clin Invest.* 2000;105(12):1827-32.

38. Tschop M, and Heiman ML. Rodent obesity models: an overview. *Experimental and clinical endocrinology & diabetes : official journal, German Society of Endocrinology [and] German Diabetes Association.* 2001;109(6):307-19.
39. Burcelin R, Crivelli V, Dacosta A, Roy-Tirelli A, and Thorens B. Heterogeneous metabolic adaptation of C57BL/6J mice to high-fat diet. *Am J Physiol Endocrinol Metab.* 2002;282(4):E834-42.
40. Maffei M, Halaas J, Ravussin E, Pratley RE, Lee GH, Zhang Y, et al. Leptin levels in human and rodent: measurement of plasma leptin and ob RNA in obese and weight-reduced subjects. *Nat Med.* 1995;1(11):1155-61.
41. Greenhill C. Celastrol identified as a leptin sensitizer and potential novel treatment for obesity. *Nat Rev Endocrinol.* 2015;11(8):444.
42. Varela L, and Horvath TL. Leptin and insulin pathways in POMC and AgRP neurons that modulate energy balance and glucose homeostasis. *EMBO reports.* 2012;13(12):1079-86.
43. Paeger L, Pippow A, Hess S, Paehler M, Klein AC, Husch A, et al. Energy imbalance alters Ca(2+) handling and excitability of POMC neurons. *eLife.* 2017;6.
44. Newton AJ, Hess S, Paeger L, Vogt MC, Fleming Lascano J, Nillni EA, et al. AgRP innervation onto POMC neurons increases with age and is accelerated with chronic high-fat feeding in male mice. *Endocrinology.* 2013;154(1):172-83.
45. Jo YH, Su Y, Gutierrez-Juarez R, and Chua S, Jr. Oleic acid directly regulates POMC neuron excitability in the hypothalamus. *J Neurophysiol.* 2009;101(5):2305-16.
46. Hill JW, Williams KW, Ye C, Luo J, Balthasar N, Coppari R, et al. Acute effects of leptin require PI3K signaling in hypothalamic proopiomelanocortin neurons in mice. *J Clin Invest.* 2008;118(5):1796-805.
47. Williams KW, Margatho LO, Lee CE, Choi M, Lee S, Scott MM, et al. Segregation of acute leptin and insulin effects in distinct populations of arcuate proopiomelanocortin neurons. *J Neurosci.* 2010;30(7):2472-9.
48. Williams KW, Sohn JW, Donato J, Jr., Lee CE, Zhao JJ, Elmquist JK, et al. The acute effects of leptin require PI3K signaling in the hypothalamic ventral premammillary nucleus. *J Neurosci.* 2011;31(37):13147-56.
49. Sohn JW, and Williams KW. Functional heterogeneity of arcuate nucleus pro-opiomelanocortin neurons: implications for diverging melanocortin pathways. *Mol Neurobiol.* 2012;45(2):225-33.
50. He Z. GY, Alhadeff AL, Castorena C, Huang Y, Lieu L, Afrin S, Sun J, Betley JN, Guo H, Williams KW. Cellular and synaptic reorganization of arcuate NPY/AgRP and POMC neurons after Exercise Article reference. *Mol Metab.* 2018.
51. Dennison CS, King CM, Dicken MS, and Hentges ST. Age-dependent changes in amino acid phenotype and the role of glutamate release from hypothalamic proopiomelanocortin neurons. *J Comp Neurol.* 2016;524(6):1222-35.
52. Jones GL, Wittmann G, Yokosawa EB, Yu H, Mercer AJ, Lechan RM, et al. Selective Restoration of Pomc Expression in Glutamatergic POMC Neurons: Evidence for a Dynamic Hypothalamic Neurotransmitter Network. *eNeuro.* 2019;6(2).
53. Lieu L, Chau D, Afrin S, Dong Y, Alhadeff AL, Betley JN, et al. Effects of metabolic state on the regulation of melanocortin circuits. *Physiol Behav.* 2020;224:113039.
54. Xu J, Bartolome CL, Low CS, Yi X, Chien CH, Wang P, et al. Genetic identification of leptin neural circuits in energy and glucose homeostases. *Nature.* 2018;556(7702):505-9.
55. Zhu C, Jiang Z, Xu Y, Cai ZL, Jiang Q, Xu Y, et al. Profound and redundant functions of arcuate neurons in obesity development. *Nat Metab.* 2020;2(8):763-74.

56. Vong L, Ye C, Yang Z, Choi B, Chua S, Jr., and Lowell BB. Leptin Action on GABAergic Neurons Prevents Obesity and Reduces Inhibitory Tone to POMC Neurons. *Neuron.* 2011;71(1):142-54.
57. Steinert RE, Feinle-Bisset C, Asarian L, Horowitz M, Beglinger C, and Geary N. Ghrelin, CCK, GLP-1, and PYY(3-36): Secretory Controls and Physiological Roles in Eating and Glycemia in Health, Obesity, and After RYGB. *Physiol Rev.* 2017;97(1):411-63.
58. Ferrario CR, Labouebe G, Liu S, Nieh EH, Routh VH, Xu S, et al. Homeostasis Meets Motivation in the Battle to Control Food Intake. *J Neurosci.* 2016;36(45):11469-81.
59. Essner RA, Smith AG, Jamnik AA, Ryba AR, Trutner ZD, and Carter ME. AgRP Neurons Can Increase Food Intake during Conditions of Appetite Suppression and Inhibit Anorexigenic Parabrachial Neurons. *J Neurosci.* 2017;37(36):8678-87.
60. Chen Y, Lin YC, Kuo TW, and Knight ZA. Sensory detection of food rapidly modulates arcuate feeding circuits. *Cell.* 2015;160(5):829-41.
61. Betley JN, Xu S, Cao ZFH, Gong R, Magnus CJ, Yu Y, et al. Neurons for hunger and thirst transmit a negative-valence teaching signal. *Nature.* 2015;521(7551):180-5.
62. Yang L, and McKnight GS. Hypothalamic PKA regulates leptin sensitivity and adiposity. *Nat Commun.* 2015;6:8237.
63. Burnett CJ, Li C, Webber E, Tsaousidou E, Xue SY, Bruning JC, et al. Hunger-Driven Motivational State Competition. *Neuron.* 2016;92(1):187-201.
64. Aponte Y, Atasoy D, and Sternson SM. AGRP neurons are sufficient to orchestrate feeding behavior rapidly and without training. *Nat Neurosci.* 2011;14(3):351-5.
65. Krashes MJ, Shah BP, Madara JC, Olson DP, Strochlic DE, Garfield AS, et al. An excitatory paraventricular nucleus to AgRP neuron circuit that drives hunger. *Nature.* 2014;507(7491):238-42.
66. Zhan C, Zhou J, Feng Q, Zhang JE, Lin S, Bao J, et al. Acute and long-term suppression of feeding behavior by POMC neurons in the brainstem and hypothalamus, respectively. *J Neurosci.* 2013;33(8):3624-32.
67. Berglund ED, Vianna CR, Donato J, Jr., Kim MH, Chuang JC, Lee CE, et al. Direct leptin action on POMC neurons regulates glucose homeostasis and hepatic insulin sensitivity in mice. *J Clin Invest.* 2012;122(3):1000-9.
68. Caron A, Dungan Lemko HM, Castorena CM, Fujikawa T, Lee S, Lord CC, et al. POMC neurons expressing leptin receptors coordinate metabolic responses to fasting via suppression of leptin levels. *Elife.* 2018;7.
69. Konner AC, Janoschek R, Plum L, Jordan SD, Rother E, Ma X, et al. Insulin action in AgRP-expressing neurons is required for suppression of hepatic glucose production. *Cell Metab.* 2007;5(6):438-49.
70. Deng Y, Wang ZV, Tao C, Gao N, Holland WL, Ferdous A, et al. The Xbp1s/GalE axis links ER stress to postprandial hepatic metabolism. *J Clin Invest.* 2013;123(1):455-68.
71. Henry FE, Sugino K, Tozer A, Branco T, and Sternson SM. Cell type-specific transcriptomics of hypothalamic energy-sensing neuron responses to weight-loss. *eLife.* 2015;4.
72. Wang D, He X, Zhao Z, Feng Q, Lin R, Sun Y, et al. Whole-brain mapping of the direct inputs and axonal projections of POMC and AgRP neurons. *Frontiers in neuroanatomy.* 2015;9:40.
73. Burke LK, Ogunnowo-Bada E, Georgescu T, Cristiano C, de Morentin PBM, Valencia Torres L, et al. Lorcaserin improves glycemic control via a melanocortin neurocircuit. *Mol Metab.* 2017;6(10):1092-102.
74. D'Agostino G, Lyons D, Cristiano C, Lettieri M, Olarte-Sanchez C, Burke LK, et al. Nucleus of the Solitary Tract Serotonin 5-HT2C Receptors Modulate Food Intake. *Cell Metab.* 2018;28(4):619-30 e5.

75. Stoss H, Pesch HJ, Pontz B, Otten A, and Spranger J. Wolcott-Rallison syndrome: diabetes mellitus and spondyloepiphyseal dysplasia. *European journal of pediatrics.* 1982;138(2):120-9.
76. Harding HP, Zeng H, Zhang Y, Jungries R, Chung P, Plesken H, et al. Diabetes mellitus and exocrine pancreatic dysfunction in perk-/- mice reveals a role for translational control in secretory cell survival. *Mol Cell.* 2001;7(6):1153-63.
77. Senee V, Vattem KM, Delepine M, Rainbow LA, Haton C, Lecoq A, et al. Wolcott-Rallison Syndrome: clinical, genetic, and functional study of EIF2AK3 mutations and suggestion of genetic heterogeneity. *Diabetes.* 2004;53(7):1876-83.
78. Delepine M, Nicolino M, Barrett T, Golamaully M, Lathrop GM, and Julier C. EIF2AK3, encoding translation initiation factor 2-alpha kinase 3, is mutated in patients with Wolcott-Rallison syndrome. *Nat Genet.* 2000;25(4):406-9.
79. Stoy J, Edghill EL, Flanagan SE, Ye H, Paz VP, Pluzhnikov A, et al. Insulin gene mutations as a cause of permanent neonatal diabetes. *Proc Natl Acad Sci U S A.* 2007;104(38):15040-4.
80. Wei J, Sheng X, Feng D, McGrath B, and Cavener DR. PERK is essential for neonatal skeletal development to regulate osteoblast proliferation and differentiation. *Journal of cellular physiology.* 2008;217(3):693-707.
81. Iida K, Li Y, McGrath BC, Frank A, and Cavener DR. PERK eIF2 alpha kinase is required to regulate the viability of the exocrine pancreas in mice. *BMC Cell Biol.* 2007;8:38.
